# Differences in reported sepsis incidence according to study design: a literature review

**DOI:** 10.1186/s12874-016-0237-9

**Published:** 2016-10-12

**Authors:** Saga Elise Mariansdatter, Andreas Halgreen Eiset, Kirstine Kobberøe Søgaard, Christian Fynbo Christiansen

**Affiliations:** Department of Clinical Epidemiology, Aarhus University Hospital, Aarhus, Denmark

**Keywords:** Sepsis, Severe sepsis, SIRS, Septicaemia, Method, Incidence, Epidemiology, Review

## Abstract

**Background:**

Sepsis and severe sepsis are common conditions in hospital settings, and are associated with high rates of morbidity and mortality, but reported incidences vary considerably. In this literature review, we describe the variation in reported population-based incidences of sepsis and severe sepsis. We also examine methodological and demographic differences between studies that may explain this variation.

**Methods:**

We carried out a literature review searching three major databases and reference lists of relevant articles, to identify all original studies reporting the incidence of sepsis or severe sepsis in the general population. Two authors independently assessed all articles, and the final decision to exclude an article was reached by consensus. We extracted data according to predetermined variables, including study country, sepsis definition, and data source. We then calculated descriptive statistics for the reported incidences of sepsis and severe sepsis. The studies were classified according to the method used to identify cases of sepsis or severe sepsis: chart-based (i.e. review of patient charts) or code-based (i.e. predetermined International Classification of Diseases [ICD] codes).

**Results:**

Among 482 articles initially screened, we identified 23 primary publications reporting incidence of sepsis and/or severe sepsis in the general population. The reported incidences ranged from 74 to 1180 per 100,000 person-years and 3 to 1074 per 100,000 person-years for sepsis and severe sepsis, respectively. Most chart-based studies used the Bone criteria (or a modification hereof) and Protein C Worldwide Evaluation in Severe Sepsis (PROWESS) study criteria to identify cases of sepsis and severe sepsis. Most code-based studies used ICD-9 codes, but the number of codes used ranged from 1 to more than 1200. We found that the incidence varied according to how sepsis was identified (chart-based vs. code-based), calendar year, data source, and world region.

**Conclusion:**

The reported incidences of sepsis and severe sepsis in the general population varied greatly between studies. Such differences may be attributable to differences in the methods used to collect the data, the study period, or the world region where the study was undertaken. This finding highlights the importance of standardised definitions and acquisition of data regarding sepsis and severe sepsis.

**Electronic supplementary material:**

The online version of this article (doi:10.1186/s12874-016-0237-9) contains supplementary material, which is available to authorized users.

## Background

Sepsis is associated with high rates of morbidity and mortality, accounting for as much as one of every two to three in-hospital deaths [1]. Notably, the mortality rates of sepsis increased during the last decade, which is in contrast to the declining rates of all other major causes of death in the US [2].

Determining the incidence of sepsis is of great interest to both clinicians and public health officials, in order to quantify the burden of the disease [3]. However, estimation of sepsis incidence is difficult, as it depends on the definition of sepsis, the method used to assess the condition, and the underlying population. Until 1992, no consensus existed on the terminology used to describe the presence and severity of sepsis, impairing comparison of studies on sepsis incidence and therapy outcomes [4]. The 1991 American College of Chest Physicians/Society of Critical Care Medicine (ACCP/SCCM) Consensus Conference addressed this issue, with the aim to create a set of criteria for identifying and assessing the severity of sepsis [5]. The consensus proposal included an introduction of the systemic inflammatory response syndrome (SIRS) criteria for early identification of sepsis, defining sepsis as 2 SIRS criteria in patients with known or suspected infection, and severe sepsis as sepsis associated with organ dysfunction, hypoperfusion, or hypotension (Table 1). Though repeatedly criticised for being too sensitive [6, 7] and of questionable prognostic value [8–10] these easily applied “Bone criteria” remained the clinical standard in many hospital guidelines even after the introduction of internationally agreed-upon, but more comprehensive, criteria [6, 11, 12]. In 2016 the definition of sepsis was updated to categorise sepsis as a life-threatening organ dysfunction caused by a dysregulated host response to infection (by The Third International Consensus Definitions for Sepsis and Septic Shock) [13].

**Table 1:** Criteria proposed to define sepsis and severe sepsis; comparison of guidelines

*Sepsis definition*			Bone *et al.*, 1992 (Sepsis-1)	Levy *et al.*, 2003 (Sepsis-2)	Dellinger *et al.*, 2013	Singer *et al.*, 2016 (Sepsis-3)		
			*Infection, documented or suspected, and at least 2 of the following (SIRS criteria):*	I*nfection, documented or suspected, and some of the following:*		*Suspected or documented infection and an acute increase of ≥2 SOFA points (a proxy for organ dysfunction)*		
General parameters		Core temperature	>38°C or <36°C	>38.3°C or <36°C		**–**		
		Heart rate	>90 bpm	>90 bpm or >2 SD above the normal value for age		**–**		
		Tachypnea	>20 breaths per minute or PaCO_2_ <32 mmHg	No specification		**–**		
		Mental status	–	Altered mental status		Glasgow coma scale:	SOFA score:	
						13-14	1	
						10-12	2	
						6-9	3	
						<6	4	
		Significant edema or positive fluid balance	–	>20 mL/kg over 24 hrs		**–**		
		Hyperglycemia in the absence of diabetes	–	Plasma glucose >120 mg/dL or >7.7 mM/L	Plasma glucose >140 mg/dL or >7.7 mM/L	**–**		
Inflammatory parameters		White blood cell count	>12,000/cu mm (leukocytosis) or <4,000/cu mm (leukopenia) or >10% immature (bands) forms	>12,000/μL (leukocytosis) or <4,000/μL (leukopenia) or Normal white blood cell count with >10% immature forms		**–**		
		Plasma C reactive protein	–	>2 SD above the normal value		**–**		
		Plasma procalcitonin	–	>2 SD above the normal value		**–**		
Hemodynamic parameters		Arterial hypotension	–	SBP <90 mmHg or MAP <70 or SBP decrease >40 mmHg in adults or <2 SD below normal for age		MAP or administration of vasopressors (μg/kg/min):	SOFA score:	
						MAP < 70 mm/Hg	1	
						dop ≤ 5 or dob (any dose)	2	
						dop > 5 or epi ≤ 0.1 or nor ≤ 0.1	3	
						dop > 15 or epi > 0.1 or nor > 0.1	4	
		Mixed venous oxygen saturation	–	>70%	–	**–**		
		Cardiac index	–	>3.5 L/min/m^2^	–	**–**		
Organ dysfunction parameters		Arterial hypoxemia	–	PaO_2_/F_IO2_ <300		PaO_2_/F_IO2_:	SOFA score:	
						<400	1	
						<300	2	
						<200 and mechanically ventilated	3	
						<100 and mechanically ventilated	4	
		Acute oliguria	–	Urine output <0.5 mL/kg/hr or 45 mmol/L for at least 2 hrs	Urine output <0.5 mL/kg/hr for at least 2 hrs despite adequate fluid resuscitation	Creatinine (mg/dl) [μmol/L] (or urine output):	SOFA score:	
						1.2–1.9 [110-170]	1	
						2.0–3.4 [171-299]	2	
						3.5–4.9 [300-440] (or < 500 mL/d)	3	
						> 5.0 [> 440] (or < 200 mL/d)	4	
		Creatinine increase	–	>0.5 mg/dL	>0.5 mg/dL or 44.2 μmol/L			
		Coagulation abnormalities	–	INR >1.5 or aPTT >60 s		**–**		
		Ileus	–	Absent bowel sounds		**–**		
		Thrombocytopenia	–	Platelet count <100 x 10^9^/L		Platelets x 103/μL:		SOFA score:
						< 150		1
						< 100		2
						< 50		3
						< 20		4
		Hyperbilirubinemia	–	Plasma total bilirubin >4 mg/dL or 70 mmol/L		Bilirubin (mg/dl) [μmol/L]:	SOFA score:	
						1.2–1.9 [> 20-32]	1	
						2.0–5.9 [33-101]	2	
						6.0–11.9 [102-204]	3	
						> 12.0 [> 204]	4	
Tissue perfusion parameters		Hyperlactatemia	–	>1 mmol/L		**–**		
		Capillary refill	–	Decreased capillary refill or mottling		**–**		
*Severe sepsis definition*			Bone *et al.*, 1992	Dellinger *et al.*, 2013		Singer *et al.*, 2016		
			*Sepsis associated with but not limited to*	*Any of the below thought to be due to the infection*		***–***		
Hypo-perfusion		Hypotension (sepsis-induced), in the absence of other causes	Systolic blood pressure < 90 mmHg or A reduction of ≥ 40 mmHg from baseline.	As defined for sepsis		**–**		
		Lactate	Lactic acidosis	Lactate above upper limit of laboratory normal		**–**		
	Organ failure	Kidney injury	Oliguria	As defined for sepsis but Creatinine > 2 mg/dL (176.8 μmol/L)		**–**		
		Acute lung injury	–	Pneumonia not the infectious source: PaO_2_/F_IO2_ < 250 or Pneumonia the infectious source: PaO_2_/F_IO2_ < 200		**–**		
		Liver injury	–	As defined for sepsis but Bilirubin > 2 mg/dL (34.2 μmol/L)		**–**		
Mental status			Acute alteration	As defined for sepsis		**–**		
Septic shock			Hypotension despite adequate fluid resuscitation along with the presence of perfusion abnormalities, as listed above.	Hypotension not reversed with fluid resuscitation.		Sepsis with persisting hypotension requiring vasopressors to maintain MAP ≥65 mmHg and having a serum lactate level >2 mmol/L (18mg/dL) despite adequate volume resuscitation.		
Multiple organ dysfunction syndrome (MODS)			Altered organ dysfunction in an acutely ill patient such that homeostasis cannot be maintained without intervention.	–		**–**		

In this review, we focus on the variation in reported incidences of sepsis and severe sepsis in the general population, and discuss the potential explanations including the use of different definitions or methods to assess sepsis.

## Methods

### Literature search and study selection

**Fig. 1:**
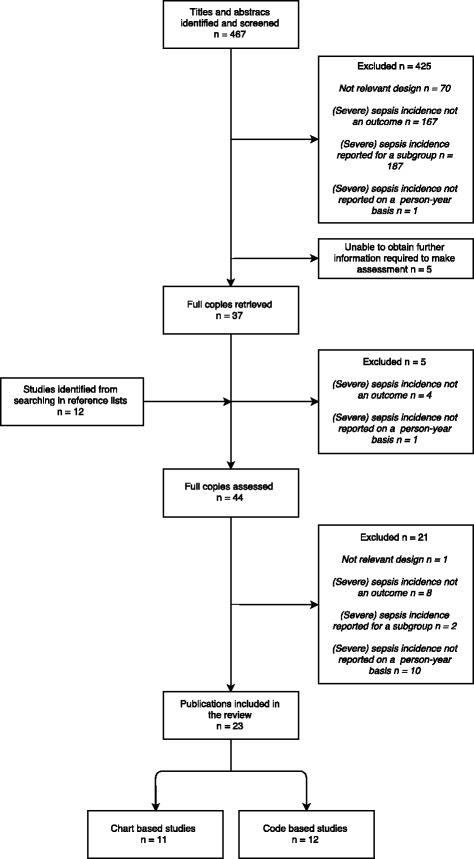
Flow chart of study selection

We included original studies with incidences of sepsis or severe sepsis in the general population (in person-years) as an outcome, published before 2016. Consequently, we excluded studies focusing on a specific subgroup of patients (e.g. neonatal sepsis, sepsis caused by a specific microbial agent), as these studies would include only a fraction of the general population as their study population. The number of excluded studies and reasons for exclusion are described in Fig. 1.

We searched PubMed (search string *(((“Sepsis/epidemiology” [Mesh]) AND (“sepsis” [Title] OR “septicaemia” [Title])) AND “incidence” [Title/Abstract]) AND “english” [Language]*), EMBASE (search string *‘sepsis’/exp OR ‘sepsis’ AND (‘epidemiology’/exp OR ‘epidemiology’ OR ‘incidence’/exp OR ‘incidence’) AND [english]/lim*) and Cochrane Library (search string *#1: MeSH descriptor: [Sepsis] explode all trees and with qualifier(s): [Epidemiology – EP] + #2: (“sepsis”:ti or “septicaemia:ti”) + #3: ”incidence”:ti,ab*).

The title and abstract of the resulting articles were screened and categorised according to predefined criteria if excluded (see section *Availability of data and materials*). All included articles – along with additional articles found in reference lists – were retrieved, read in full and excluded according to the same criteria (see Fig. 1). Two authors (SEM and AHE) performed all rounds independently; the final decision to exclude an article was reached by consensus.

Data were extracted from each study according to a predetermined list of variables (see section *Availability of data and materials*). If a study reported several incidences – e.g. for different years or applying different methodologies – each incidence measure was registered as an observation. We adapted a widely used terminology to categorise the studies according to method used to identify sepsis or severe sepsis: 1. “chart-based” including studies that identified patients by review of patient charts and 2. “code-based” including studies that identified patients using diagnostic codes [3, 14–16]. To examine regional differences in incidence of sepsis and severe sepsis each study was categorised according to World Bank region [17].

Data management and descriptive statistics were performed using R [18]. In order to examine the heterogeneity that gives rise to the differences in incidence as well as possible interactions, we produced a number of boxplots based on crude data to allow for a visual evaluation of some of the factors that influence the reported incidence. Further, we present detailed tables that allow the reader to compare the included studies. The data set, along with the R-code and codebook, are freely available (see section *Availability of data and materials*).

### International Classification of Diseases (ICD)

In the code-based studies, ICD codes were used to identify cases from discharge databases without specific information on physiological parameters. Implementation of the tenth revision of the ICD coding system (ICD-10) started in 1994 [19], but actual implementation dates vary among countries and was finally completed in the US as of October 1, 2015 [20]. Consequently, ICD-10 data was used in only two studies [21, 22]. A table with the full lists of specific sepsis codes in the ICD-9 and ICD-10 coding systems are provided as an additional file (see Additional file 1).

Below is a brief summary of the development of the guidelines used; Table 1 offers a detailed comparison of sepsis, severe sepsis, septic shock and multiple organ dysfunction syndrome.

### The 1991 ACCP/SCCM Consensus Conference guidelines

In 1992 Bone et al. proposed a standardised definition of sepsis [5]. This included an introduction of the four SIRS criteria: 1. Temperature >38 °C or <36 °C; 2. heart rate >90 beats per minute; 3. respiratory rate >20 breaths per minute or PaCO2 < 32 mmHg; and 4. white blood cell count >12,000/cu mm, <4,000/cu mm, or >10 % immature (band) forms. According to this, systemic inflammatory response syndrome (SIRS) was defined as at least two SIRS criteria, and sepsis was defined as (suspected) infection and at least two SIRS criteria. In addition it was suggested that use of the term “septicaemia” should be avoided. We will refer to this definition as the “Bone criteria”.

### International Sepsis Definitions Conference modifications

In 2003, the first Surviving Sepsis Campaign was published [6]. In an effort to increase the clinical utility, the diagnostic criteria were expanded to include other parameters, among these inflammatory, hemodynamic and tissue perfusion. It was emphasised that none of these new criteria were specific for sepsis. The latest campaign edition published in 2012 contained only minor revisions, and thus these expanded criteria have remained the recommended clinical standard [3]. However, a revised international definition of sepsis criteria has recently been published [13], in which the SIRS criteria are replaced by the sepsis-related organ failure assessment (SOFA) score [23].

## Results

Our search identified 467 articles of which 430 were excluded after screening (see Fig. 1). An additional 12 articles were identified from the reference lists of the included articles, of which five were excluded after going through the abstracts. Of 44 articles read in full 21 were excluded: 10 articles did not provide sepsis or severe sepsis incidence on a person-year basis [15, 24–32], eight articles did not report sepsis or severe sepsis incidence as an outcome [33–40], two articles reported sepsis or severe sepsis incidence for a subgroup of patients [41, 42] and one article did not use a relevant design to compute sepsis and severe sepsis incidences [43]. Thus, we included a total of 23 articles: 11 chart-based and 12 code-based studies. Summaries of the included studies can be found in Tables 2 and 3.

**Table 2:** Chart-based studies of sepsis and severe sepsis incidence in the general population

	Padkin, 2003 [54]	Finfer, 2004 [48]	Brun-Buisson, 2004 [45]	Harrison, 2006 [53]	Esteban, 2007 [47]	Karlsson, 2007 [50]	Blanco, 2008 [44]	Vesteinsdottir, 2011 [52]	Davis, 2011 [46]	Nygard, 2014 [51]	Henriksen, 2015 [49]
Country/region	England, Wales and Northern Ireland	Australia and New Zealand	France	England, Wales and Northern Ireland	Madrid, Spain	Finland	Castilla y Leòn Region, Spain	Iceland	Northern territory, Australia	Norway	Denmark
Setting	91 ICUs	23 ICUs	206 ICUs	172 ICUs	3 hospitals	24 ICUs/11 hospitals	11 ICUs	3 ICUs	1 hospital	3 ICUs	1 ED
Study population	56,673	5,878	3,738	343,860	15,852	4,500	2,619	1,524	15,963	NA	8,358
Number of cases (sepsis/severe sepsis)	NA/15,362	NA/691	NA/621	NA/92,672	702/199	NA/472	NA/246	NA/115	1,191/272	NA/220	621/1,071
Study duration	1995–2000	3 months	2 weeks	10 year	4 months	4 months /4 days	6 months	1 year	1 year	1 year	1 year
Exclusion criteria	<16 years, readmissions, sepsis not present within 24 h from admission	<15 years		<16 years, readmissions, sepsis not present within 24 h from admission	< 18 years	< 18 years, readmissions	<18 years	< 18 years, readmissions, sepsis not present on admission	< 15 years	< 15 years, severe sepsis not present within 24 h from admission, transferred with diagnosis of severe sepsis	< 15 years, readmissions, immediately preceding hospitalisation
Sepsis inclusion criteria	PROWESS	Bone criteria	Bone criteria	PROWESS	Bone criteria	Bone criteria	Bone criteria	Bone criteria	PROWESS	Bone criteria	Bone criteria
Organ failure inclusion criteria	Modified PROWESS	Modified PROWESS	SOFA score ≥3	Modified PROWESS	MODS score >2	SOFA score ≥3	Modified PROWESS	Modified Bone criteria	PROWESS	Modified Levy et al.	Protocol specified criteria
Calendar year^i^	1997^ii^	1999	2001	1996; 2003	2003	2005	2002	2009	2008	2008	2011
Sepsis incidence 100.000 person yrs^−1^	–	–	–	–	367	–	–	–	1,180	–	265
Severe sepsis incidence 100.000 person yrs^−1^	51	77	95	46; 66	104	38	25	48	130	50	457

Characteristics of chart based studies of sepsis and severe sepsis incidence extrapolated to the general population. i) If study is conducted in two consecutive calendar years the last year is reported. ii) If full data were not available for 1997, the closest full year’s data were used. *Abbreviations*: −, not calculated; *ED* emergency department, *hrs* hours, *ICU* intensive care unit, *MODS* multiple organ dysfunction syndrome, *NA* not available, *PROWESS* Protein C Worldwide Evaluation in Severe Sepsis, *SOFA*, sequential organ failure assessment, *yrs* years old

**Table 3:** Code-based studies of sepsis and severe sepsis incidence in the general population

	CDC, 1990 [57]	Angus, 2001 [14]	Martin, 2003 [63]	Flaatten, 2004 [21]	Dombrovskiy, 2005 [60]	Esper, 2006 [61]	Dombrovskiy, 2007 [59]	Shen, 2010 [56]	Wilhelms, 2010 [22]	Kumar, 2011 [62]	Lagu, 2012 [16]	Chen, 2013 [58]
Country/Region	USA	USA (7 states)	USA	Norway	New Jersey, USA	USA	USA	Taiwan	Sweden	USA	USA	Taiwan
Coding system	ICD-9	ICD-9	ICD-9	ICD-10	ICD-9	ICD-9	ICD-9	ICD-9	ICD-9/10^vi^	ICD-9	ICD9	ICD9
Data source	NHDS	Constructed database	NHDS	NPR	New Jersey SID	NHDS	NIS	NHIRD	SHDR	NIS	NIS	NHIRD
Study population	NA	6,621,559	NA	700,107	7,364,550	NA	NA	201,657^iv^ 200,000^v^	2,024,793	NA	NA	NA
Number of cases (sepsis/severe sepsis)	NA	NA/192,980	NA	6665/NA	24,765 - 30,081/8096 - 13,453	NA	NA	NA/7531^iv^ NA/5258^v^	NA/37,990^vii^ NA/27,655^vii^ NA/12,512^vii^	NA	NA	NA/40,856 - 116,749
Exclusion criteria	<1 year			Neonate sepsis	<18 years			Previous episode of severe sepsis	Neonate sepsis, previous episode of severe sepsis	<18 years	<18 years	
Internal validation	No	Yes	Yes	No	No	(Yes)^ii^	No	Yes	No	No	No	No
Calendar year	1979; 1987	1995	1979; 2000	1999	1995–2002	1979; 2003	1993–2003	1997; 2006	1987; 2005	2000; 2007	2007	1997-2008
Sepsis incidence 100.000 person yrs^−1^	74; 176	–	83; 240^i^	149	–	83; 275^i^	–	–	–	–	–	-
Severe sepsis incidence 100.000 person yrs^−1^	–	300	–	–	135–208	–	65–135	153; 359^iii,iv^ 135; 217^iii,v^	10; 35^vii^ 25; 43^vii^ 3; 13^vii^	143; 343	1074^viii^ 303^viii^	188 - 507

Characteristics of code based studies of sepsis and severe sepsis incidence extrapolated to the general population. i) Age-standardized to fit the population distribution in the 2000 U.S. consensus. ii) Method validated by Martin et al. iii) Age-standardized using 2000 world population reported by WHO as standard. iv) No exclusion criteria. v) Exclusion criteria as stated. vi) Discharge diagnoses were classified according to ICD-9 until the end of 1996. These were translated into ICD-10 for the methods of Angus et al. and Martin et al. vii) Using the method proposed in Angus et al., Flaaten et al. (time of incidence measure: 1997; 2005) and Martin et al., respectively. viii) Using the method proposed in Angus et al. and Dombrovskiy et al., respectively. *Abbreviations*: −, not calculated; *SHDR* Swedish hospital discharge register, *NA* not available, *NHDS* national hospital discharge survey (USA), *NHIRD* national health insurance research (Taiwan), *NIS* nationwide inpatient sample (USA), *NPR* Norwegian patient register; yrs, years

### Chart-based studies

Nine studies [44–52] screened patients according to pre-defined criteria for sepsis and/or severe sepsis; two studies [53, 54] analysed previously collected data. One chart-based study on severe sepsis reported incidences for several years. Most chart-based studies used the Bone criteria (or a modification hereof) and Protein C Worldwide Evaluation in Severe Sepsis (PROWESS) study criteria to identify cases of sepsis and severe sepsis (Table 2). For organ dysfunction definitions, adaptations of the PROWESS study criteria [55] were the most frequently used (see Additional file 2 for a detailed description).

### Code-based studies

Three code-based studies applied different algorithms to the same data set [16, 22, 56] while three and six code-based studies reported several years' observations of sepsis and severe sepsis incidences, respectively [22, 56–63] (Table 3).

Most code-based studies used ICD-9, though there was great diversity in what and how many codes were used, ranging from 1 to more than 1200 (see Additional file 3).

Three code-based studies used the Bone criteria for validation: Angus et al. and Shen et al. [14, 56] used the combination of ICD codes defined in their methods applied to an alternate cohort and a randomly selected database sample, respectively, while Martin et al. [63] compared only the ICD-9 codes specific for septicaemia to a chart-based method. In general, there was a high degree of agreement between patients identified using ICD codes and patients identified by the Bone criteria, respectively. However, Angus et al. did find that their ICD codes generated higher incidences than what was found for the reference cohort using clinical and physiologic data [14].

### Sepsis and severe sepsis incidence in the general population

**Fig. 2:**
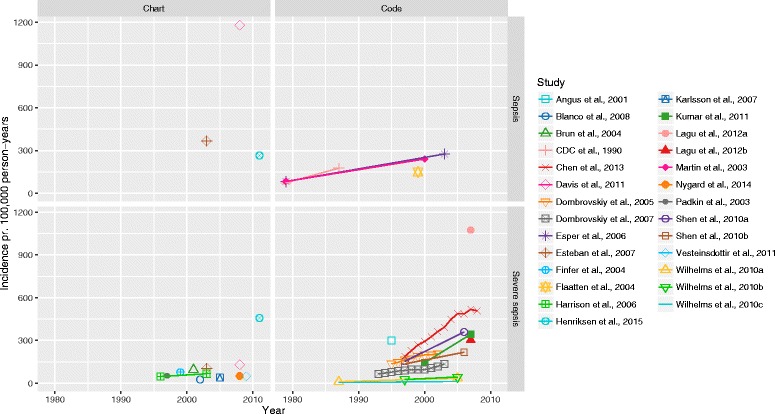
Incidence over time. Each study is identified by colour and symbol

**Fig. 3:**
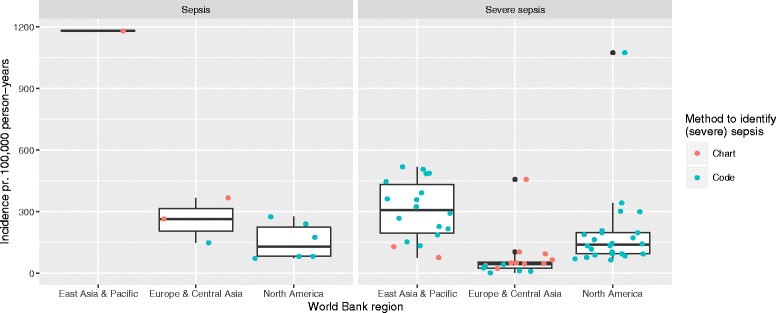
Boxplot of the incidence of sepsis and severe sepsis stratified on World Bank region. The figure gives a crude estimate of the median, the interquartile range (IQR), and the highest and lowest value within 1.5 × IQR. Data beyond the end of the whiskers are plotted as black points. Points represent single observations that contribute data to the estimate; colours indicate whether the study is chart- or code-based

Overall, we found great variation in incidence both between and across methods used to identify sepsis and severe sepsis, ranging from 74 to 1180 per 100,000 person-years and 3 to 1074 per 100,000 person-years, respectively. The incidence of both sepsis and severe sepsis increased over time (Fig. 2). When stratifying on method used to identify sepsis, we found that chart-based studies in general reported a higher incidence of sepsis than the code-based studies, whereas the opposite was the case for severe sepsis. There was a great diversity in the data source used: studies including patients from all wards in the hospital (”Hospital wide”) found the highest sepsis incidence whereas studies only including patients from intensive care units (ICUs) found a relatively low severe sepsis incidence (see Additional files 4 and 5). Stratifying on World Bank region, we found the lowest sepsis incidence in North America and the lowest severe sepsis incidence in the Europe & Central Asia region; in both cases the incidence was highest in the East Asia & Pacific region (Fig. 3). In addition, we examined for interaction between calendar year, World Bank region and method (plots not shown). While we did find interaction with calendar year for both World Bank region and chart/code based studies, there was a consistent trend in the rise of incidence. The interaction of method and World Bank region can be seen in Fig. 3.

## Discussion

In this literature review, we found that the reported incidence of sepsis and severe sepsis in the general population varied greatly between the included studies. We compared the methods used and the demographic characteristics of the studied populations. We found that the variation may in part be attributable to whether a chart-based or a code-based method was used, differences in the criteria used for identifying cases of sepsis or severe sepsis within these groups, year of incidence measure, and the World Bank region in which the study was conducted.

In most chart-based studies on severe sepsis incidence, cases were identified in ICUs only. Such selection might introduce bias towards a lower incidence because patients that fulfil the criteria for severe sepsis but did not need ICU care were excluded. Indeed, these studies did on average find a lower incidence of severe sepsis than studies with other inclusion criteria. However, the chart-based study by Karlsson et al. [50] included admissions to both ICUs and other hospital wards, and still found an incidence of severe sepsis in adults much lower than what was found within a similar time period in the code-based studies of Dombrovskiy et al. [60] and Kumar et al. [62]. This indicates that other factors play an important role for the observed differences in incidence between chart- and code-based studies, and the question is whether these very different approaches are even comparable. Wilhelms et al. [22] addressed this by applying the methods of Angus et al.*,* Flaatten, and Martin et al. [14, 21, 63] to the same database. Notably, Wilhelms found that the methods identified very different patient cohorts with little overlap, questioning whether the ICD codes correspond to the clinical definition of severe sepsis. As mentioned previously, Angus et al. did indeed find that their criteria generated higher incidences than the Bone criteria, but most of the code-based studies did not explore the clinical characteristics of identified cases, even though many codes not specific for sepsis were used. In a US study by Gaieski et al. [15], the methods of Angus et al., Wang et al., Dombrovskiy et al., and Martin et al. [2, 14, 59, 63], were all applied to a cohort identified using the Nationwide Inpatient Sample (NIS) database, which was also used in some of the included code-based studies [16, 59, 62]. The incidences found using each of these methods were compared to the incidence found using the specific ICD-9 sepsis codes only. Apart from finding that these methods led to very different estimates of severe sepsis, the authors also found that only between 14 % (Wang, Angus) and 48 % (Dombrovskiy) of severe sepsis cases had been assigned the ICD-9 severe sepsis code (995.92).

The increase found in both sepsis and severe sepsis incidence over the years could be due to an actual increase caused by factors such as increasing prevalences of co-morbidities in the general population, a change in the population demographics with more elderly, use of intravenous accesses or other predisposing factors for sepsis. However, an increased clinical and political awareness of sepsis, as pursued by the Surviving Sepsis campaigns, or perhaps a change in coding practice could also lead to higher estimates [64]. Probably, the increase in reported incidences is caused by a combination of several or all of these. As recently suggested, an automatic epidemiological surveillance system based on electronic health records for patients with sepsis, may give better estimates for both sepsis incidence and mortality [65].

When stratifying on World Bank region, we found a variation in incidences of both sepsis and severe sepsis. Remarkably, the incidence of sepsis was generally lower in the North America region compared to Europe & Central Asia, whereas the opposite was the case for severe sepsis. These differences may arise from differences in coding practice and the related economic incentive, and access to hospital and ICU care. The study by Wilhelms et al. [22] supports this observation: When reproducing the studies by Angus et al. [14] and Martin et al. [63] on a Swedish cohort they find remarkably lower incidences than was reported for the studies set in North America.

The relatively low number of studies on sepsis and severe sepsis incidence after stratifying on code-based or chart-based studies limits our review. Also, the great heterogeneity of the included studies, such as the number and type of codes used to define sepsis and severe sepsis in the code-based studies, may not only give rise to major differences in outcome but also impedes direct comparison, as the studies differs from each other by several variables.

The importance of reaching a greater consistency in the definition of sepsis and severe sepsis used in epidemiological studies has been commented by Singer et al. [13], following the third international sepsis definition consensus conference, and recommendations are given for both clinical identification of sepsis as well as ICD coding. If these recommendations are successfully implemented worldwide, this may offer a more simple and intuitive approach to diagnosis of sepsis and septic shock. This approach, together with the proposed recommendations for registration of the condition, may not only lead to a more prompt recognition of sepsis, but also enable a higher consistency for epidemiological studies reporting sepsis incidence.

## Conclusion

The reported incidence of sepsis and severe sepsis in the general population varies greatly between studies. In this literature review, we present a detailed systematic examination of all original studies reporting the incidence of sepsis or severe sepsis in the general population as a main outcome. We find that the methods used differ between the studies to a degree that greatly hampers the inference about any variable's impact on the incidence. This highlights the importance of standardised definitions and acquisition of data regarding sepsis and severe sepsis.

## Additional files

Additional file 1: The specific ICD codes for sepsis in the 9th and 10th revision. (PDF 69 kb)
Additional file 2: Comparison of the different criteria used to define organ dysfunction in the chart-based studies. (PDF 120 kb)
Additional file 3: The ICD-9 codes used in the included studies (except study by Flaaten in which ICD-10 codes were used). (PDF 139 kb)
Additional file 4: Boxplot of the incidence of sepsis stratified by protocol used to identify cases and on data source. (PDF 6 kb)
Additional file 5: Boxplot of the incidence of severe sepsis stratified by protocol used to identify cases and on data source. (PDF 10 kb)

## Abbreviations

ACCP/SCCM: American College of Chest Physicians/Society of Critical Care Medicine
ICD: International Classification of Diseases
ICU: Intensive care unit
PROWESS: Protein C Worldwide Evaluation in Severe Sepsis
SIRS: Systemic inflammatory response syndrome
