# Supplementary material for: Differences in reported sepsis incidence according to study design: a literature review
Source: BMC Med Res Methodol. 2016 Oct 12;16:137. doi: 10.1186/s12874-016-0237-9 (PMC5062833; doi:10.1186/s12874-016-0237-9)
Supplement: Additional file 1: — The specific ICD codes for sepsis in the 9th and 10th revision. (PDF 69 kb) [file 12874_2016_237_MOESM1_ESM.pdf]

Specific ICD codes for sepsis in the 9th and 10th revision.

ICD-9	038 Septicemia<br>038.0 Streptococcal septicemia<br>038.1 Staphylococcal septicemia<br>.10… unspecified<br>.11 Methicillin susceptible Staphylococcus aureus septicemia<br>.12 Methicillin resistant Staphylococcus aureus septicemia<br>.19 Other staphylococcal septicemia<br>038.2 Pneumococcal septicemia [Streptococcus pneumoniae septicemia]<br>038.3 Septicemia due to anaerobes<br>038.4 Septicemia due to other gram-negative organisms<br>.40 Septicemia due to gram-negative organism, unspecified<br>.41 Septicemia due to Hemophilus influenzae [H. influenzae]<br>.42 Septicemia due to Escherichia coli [E. coli]<br>.43 Septicemia due to Pseudomonas<br>.44 Septicemia due to Serratia<br>.49 Other septicemia due to gram-negative organisms<br>038.8 Other specified septicemias<br>038.9 Unspecified septicemia<br><br>995.9 Systemic inflammatory response syndrome (SIRS)<br>995.90 Systemic inflammatory response syndrome, unspecified<br>995.91 Sepsis<br>995.92 Severe sepsis<br>785.52 Septic shock<br>995.93 Systemic inflammatory response syndrome due to noninfectious process without acute organ dysfunction<br>995.94 Systemic inflammatory response syndrome due to noninfectious process with acute organ dysfunction
ICD-10	A02.1 Salmonella sepsis<br>A22.7 Anthrax sepsis<br>A26.7 Erysipelothrix sepsis<br>A32.7 Listerial sepsis<br>A40 Streptococcal sepsis<br>40.0 Sepsis due to Streptococcus, grp. A<br>40.1 Sepsis due to Streptococcus, grp. B

40.3 Sepsis due to *Streptococcus pneumoniae*

A41 Other sepsis

A41.0 Sepsis due to *Sthapylococcus aureus*

.01 Sepsis due to Methicillin susceptible *Staphylococcus aureus*

.02 Sepsis due to Methicillin resistant *Staphylococcus aureus*

A41.1 Sepsis due to other specified Staphylococcus

A41.2 Sepsis due to unspecified Staphylococcus

A41.3 Sepsis due to *Hemophilus influenzae*

A41.4 Sepsis due to anaerobes

A41.5 Sepsis due to other Gram-negative organisms

.50 Gram-negative sepsis, unspecified

.51 Sepsis due to *Escherichia coli [E. coli]*

.52 Sepsis due to *Pseudomonas*

.53 Sepsis due to *Serratia*

.54 Other Gram-negative sepsis

A41.8 Other specified sepsis

.81 Sepsis due to *Entercoccus*

.82 Other specified sepsis

A41.9 Sepsis, unspecified

- Use additional code R65.20 if desired to identify severe sepsis without septic shock
- Use additional code R65.21 if desired to identify severe sepsis with septic shock

A42.7 Actinomycotic sepsis

B37.7 Candidal sepsis
