# Supplementary material for: Differences in reported sepsis incidence according to study design: a literature review
Source: BMC Med Res Methodol. 2016 Oct 12;16:137. doi: 10.1186/s12874-016-0237-9 (PMC5062833; doi:10.1186/s12874-016-0237-9)
Supplement: Additional file 2: — Comparison of the different criteria used to define organ dysfunction in the chart-based studies. (PDF 120 kb) [file 12874_2016_237_MOESM2_ESM.pdf]

Comparison of the different criteria used to define organ dysfunction in the chart-based studies

	**Padkin, 2003** ***and*** **Harrison, 2006**	**Finfer, 2004**	**Brun-Buisson, 2004** ***and*** **Karlsson, 2007**	**Esteban, 2007**	**Blanco, 2008**	**Vesteinsdottir, 2011**	**Davis, 2011**	**Nygard, 2014**	**Henriksen, 2015**
	Modified PROWESS. At least one is present during the first 24 hrs:	Modified PROWESS. At least one is induced by sepsis:	SOFA[iv] ≥3. For each organ system this is achieved by:	MODS[iii] >2. For each organ system this is achieved by:	Modified PROWESS. At least one is induced by sepsis and present for 24 hrs:	Modified Bone criteria. At least one present during the first 24 hrs of intensive care:	PROWESS. At least one present during the first 24 hrs:	Modified criteria as presented in Levy *et al.* Some of the following:	Protocol specified criteria. One of the following:
**Cardio-vascular**	SBP <90 mm Hg or MAP <70 mm Hg or the use of vasoactive drugs for 1 hr in the first 24 hrs	An arterial SBP of ≤90 mmHg or MAP ≤70 mmHg for at least 1 h despite adequate fluid resuscitation[i], adequate intravascular volume status, and/or need for vasopressors[ii] to maintain systolic blood pressure >90 mmHg or MAP >70 mmHg for >1 h	Hypotension in spite of adrenergic agents administered for at least one hour: dopamine or epinephrine, or norepinephrine.	PAR ≥20.1	Arterial SBP of ≤90 mmHg or MAP ≤70 mmHg for at least 1 h. despite adequate fluid resuscitation[i], adequate intravascular volume status, and/or need for vasopressors[ii] to maintain systolic blood pressure >90 mmHg or MAP >70 mmHg for >1 h	SBP <90 mmHg or MAP <65 mmHg	Arterial SBP <90 mm Hg or MAP <70 mm Hg for at least 1 hr despite adequate fluid resuscitation, adequate intravascular volume status or the use of vasopressors	SBP < 90 mmHg or MAP decrease >40 mmHg	-
**CNS**	-	-	-	GCS ≤9	Encephalopathy with GCS <13 without sedation unexplained by other causes	Altered mental status	-	Altered mental status	GCS ≤14 recorded on admission or any kind of CNS affection recorded in the electronic patient records on admission if GCS was missing. If pre-existing dementia was known, then proof of deterioration from the preceding usual state was required.
**Haematological**	Platelet count <80*10^9/L	Platelet count of <80*10^9/L or a 50% decrease from the highest value in the previous 3 days	Platelet count <50*10^9/L	Platelet count ≤50*10^9/L	Platelet count <80*10^9/L or decrease of 50% in the 3 previous days	Platelet count <80*10^9/L, or aPTT >60 seconds	Platelet count <80*10^9/L or decrease of 50% in the 3 previous days	Platelet count <100*10^9/L or a 50 % reduction during the last 3 days	Platelet count <101*10^9/L and earlier platelet count >100*10^9/L or never previously registered or INR >1.59 and earlier INR <1.60 or never previously registered, without the use of Warfarin

**Hepatic**	-	-	Bilirubin ≥102 µmol/L	Bilirubin ≥121 µmol/L	Bilirubin >3 mg/dl or prothrombin time <50% related to a hepatic cause	Bilirubin >70 µmol/L	-	Bilirubin >70 µmol/L	Bilirubin >42 µmol/L and earlier bilirubin <43 µmol/L or never previously registered
**Metabolic**	Base deficit 5.0 mmol/L	Unexplained metabolic acidosis defined by (a) pH <7.30 of metabolic origin or base deficit >5.0 mmol/L, or (b) A plasma lactate level >1.5 times the upper limit of normal for the reporting lab	-	-	Unexplained metabolic acidosis: (pH <7.30 or base excess ≤-5 mmol/L) associated with an arterial lactate concentration ≥ 2 mmol/l with no other apparent cause	Metabolic acidosis: pH <7.30, or elevated lactate: >2.0 mmol/L	Unexplained metabolic acidosis: (pH <7.30 or base excess ≤-5 mmol/L) in association with a lactate level >1.5 times the upper limit of the normal value for the reporting laboratory	pH <7.30 and lactate >4.0 mmol/L	pH <7.31 or lactate >2.5 mmol/L
**Renal**	Mean hourly urine output 0.5 mL kg body weight in the first 24 hrs in ICU or for the duration of stay if 24 hrs in ICU. If on chronic renal replacement therapy, admission needed to meet another organ dysfunction criteria	Average urine output <0.5 mL/kg hr^{-1} for a 4-hr period, despite adequate fluid resuscitation as described above. In the presence of pre-existing impairment of renal function (defined as serum creatinine concentration >2 times the upper limit of the normal reference range for your institution prior to the onset of sepsis), the patient would need to meet one of the other organ-failure criteria	Creatinine ≥300 µmol/L, or urine output <500mL/day	Creatinine ≥351 µmol/L	Diuresis <0.5 mL/kg/hr despite adequate blood volume, or creatinine >1.9 mg/dL	Diuresis <0.5 mL/kg/hr for at least two hours, or acute creatinine increase ≥170 µmol/L, or doubling of baseline creatinine value	Diuresis <0.5 ml/kg/hr for one hour, despite adequate fluid resuscitation	Diuresis <0.5 ml/kg/hr or 45 mmol/L for at least 4 hrs or creatinine >50 µmol/L	Creatinine >177 µmol/L and >100 µmol/L creatinine increase from earlier creatinine or creatinine >177 µmol/L and earlier creatinine <130 µmol/L or never previously registered

**Respiratory**	PaO_2/FiO_2 ratio 250 mm Hg. If the lung is the sole organ meeting an organ dysfunction criterion and primary/secondary reason for ICU admission indicated lung infection, PaO_2/FiO_2 must be 200 mm Hg.	Presence of ARDS defined as:<br>- $PaO_2/FiO_2 \leq 200$<br>- Bilateral alveolar infiltrate on CXR<br>- Absence of cardiac cause of pulmonary oedema, i.e. PCWP <18 mmHg, or if no PAC the treating clinicians are of opinion pulmonary infiltrate is not due to increased left atrial pressure	PaO_2/FiO_2 <200 with respiratory support	$PaO_2/FiO_2 \leq 150$	Mechanical ventilation; or PaO_2/FiO_2 <250 mmHg if other organ dysfunction was present; or PaO_2/FiO_2 < 200 mmHg if only pulmonary dysfunction was present	PaO_2/FiO_2 <250	PaO_2/FiO_2 <250 mmHg if other organ dysfunction was present; or PaO_2/FiO_2 < 200 mmHg if only pulmonary dysfunction was present	SpO_2 < 90 % while breathing air	Oxygen saturation <92% recorded at arrival or arterial O_2 tension <9.75 kPa (<73 mmHg)<br>or<br>arterial O_2 saturation <0.92 if oxygen saturation was missing

i) Adequate fluid resuscitation or adequate intravascular volume is defined as one or more of the following: (a) The administration of an intravenous fluid bolus (≥500 mL of crystalloid solution, ≥20 g of albumin, or ≥200 mL of other colloids administered over 60 min or less) (b) Pulmonary arterial wedge pressure ≥12 mmHg (c) CVP ≥8 mmHg.

ii) Vasopressors defined as: (a) Dopamine >5 mg/kg $minute^{-1}$ (b) Noradrenaline, adrenaline, metaraminol, or phenylephrine by infusion at any dose.

iii) Marshall et al., “Multiple Organ Dysfunction Score: A reliable descriptor of a complex clinical outcome”, Crit Care Med, 1995.

iv) Moreno et al., “The use of maximum SOFA score to quantify organ dysfunction/failure in intensive care. Results of a prospective, multicentre study”, Intensive Care Med, 1999. Abbreviations: PROWESS, recombinant human activated protein C worldwide evaluation in severe sepsis study group; SOFA, sequential organ failure assessment; MODS, multiple organ dysfunction score; SBP, systolic blood pressure; MAP, mean arterial pressure; PAR, pressure-adjusted heart rate; GCS, Glasgow Coma Score.
