# Supplementary material for: Differences in reported sepsis incidence according to study design: a literature review
Source: BMC Med Res Methodol. 2016 Oct 12;16:137. doi: 10.1186/s12874-016-0237-9 (PMC5062833; doi:10.1186/s12874-016-0237-9)
Supplement: Additional file 3: — The ICD-9 codes used in the included studies (except study by Flaaten in which ICD-10 codes were used). (PDF 139 kb) [file 12874_2016_237_MOESM3_ESM.pdf]

ICD-9 codes used in the included studies.

	CDC, 1990	Angus, 2001*	Martin, 2003	Dombrovskiy, 2005	Esper, 2006	Dombrovskiy, 2007	Shen, 2010*	Kumar, 2011	Chen, 2013*
**Sepsis**									
Septicemia<br>038.0-038.9	•	•	•	•	•	•	•	•	•
Herpetic septicemia<br>054.5				•		•			
Bacteremia<br>790.7		•	•		•		•	•	•
Disseminated fungal infection<br>117.9		•	•		•		•	•	•
Disseminated candidal infection<br>112.5		•	•	•	•	•	•	•	•
Candidal endocarditis<br>112.81		•	•		•		•	•	•
Fungal endocarditis<br>115.04, 115.14, 115.94		•					•	•	•
Salmonella septicemia<br>003.1		•		•		•	•	•	•
Septicemic plague<br>020.2		•	•	•		•	•	•	•
Anthrax septicemia<br>022.3		•		•		•	•	•	•
Meningococcal septicemia<br>036.2		•		•		•	•	•	•
Waterhouse Friedrichson Syndrome<br>036.3		•		•		•	•	•	•
Gonococcemia<br>098.89		•		•		•	•	•	•
Sepsis due to indwelling catheter<br>996.62		•					•	•	•
Sepsis**<br>995.91				•		•			
Severe sepsis**<br>995.92				•		•		•	
Septic shock***<br>785.52						•		•	
**Severe sepsis**									
**Respiratory**									
93.90<br>Non-invasive mechanical ventilation							•		•
96.04<br>Continuous positive airway pressure	NA				NA		•		•
Ventilator management<br>96.7		•	•				•	•	•

Acute respiratory failure<br>518.81			•	•		•	•	•	•
Acute respiratory distress syndrome (ARDS)<br>518.82			•	•		•	•	•	•
ARDS after shock or trauma<br>518.85			•				•	•	•
Respiratory insufficience<br>786.09			•	•		•	•	•	•
Respiratory arrest<br>799.1			•	•		•	•	•	•
**Cardiovascular**									
Use of vasopressor agent<br>00.17								•	
Cardiac arrest<br>427.5				•		•			
Hypotension, postural<br>458.0		•	•	•		•	•		•
Hypotension, specified, not elsewhere classified<br>458.8		•	•	•		•	•	•	•
Hypotension, arterial, constitutional<br>458.9		•	•	•		•	•	•	•
Shock<br>785.50		•	•	•		•	•	•	•
Shock, cardiogenic<br>785.51		•	•	•		•	•	•	•
Septic shock****<br>785.52				•		•		•	
Shock, circulatory or septic<br>785.59		•	•	•		•	•	•	•
Hypotension, transient<br>796.3			•	•		•	•	•	•
**Renal**									
Hemodialysis<br>573.3			•				•		•
Acute glomerulonephritis<br>580			•				•		•
Acute renal failure<br>584		•	•	•		•	•	•	•
Chronic kidney disease<br>585									
Renal failure, unspecified<br>586			•				•		•
**Hepatic**									
Acute hepatic failure or necrosis<br>570		•	•	•		•	•	•	•
Hepatic encephalopathy<br>572.2			•	•		•	•	•	•
Hepatitis, septic or unspecified<br>573.3			•				•		•
Hepatic infarction<br>573.4		•		•		•	•	•	•
**Hematologic**									

Disseminated intravascular coagulation 286.2			•				•		•
Purpura fulminans 286.6		•	•	•		•	•	•	•
Acquired coagulation factor deficiency 286.7								•	
Coagulopathy 286.9		•	•	•		•	•	•	•
Thrombocytopenia, primary 287.3			•				•		•
Thrombocytopenia, secondary 287.4		•	•	•		•	•	•	•
Thrombocytopenia, unspecified 287.5		•	•	•		•	•	•	•
Neutropenia 288.0									
Abnormal coagulation profile 790.92							•		•
**Metabolic**									
Acidosis, metabolic or lactic 276.2			•				•	•	•
**Neurologic**									
Electroencephalography 89.14			•				•	•	•
Transient organic psychosis 293		•	•	•		•	•	•	•
Anoxic brain injury 348.1		•	•	•		•	•	•	•
Encephalopathy, acute 348.3		•	•	•		•	•	•	•
Coma 780.01			•	•		•	•	•	•
Altered consciousness, unspecified 780.09			•				•	•	•

** 1,286 distinct infection codes included*
*** Implemented on October 1, 2002*
**** Implemented on October 1, 2003*
***** Implemented on October 1, 2003. Only to be used with code 995.2 for severe sepsis.*
Where 4-digit codes are listed, all associated subcodes were included.
In case of discrepancies between code number and corresponding code text given by the article, the code given in this table was assigned due to *code text*.
