# Supplementary material for: Differences in reported sepsis incidence according to study design: a literature review
Source: BMC Med Res Methodol. 2016 Oct 12;16:137. doi: 10.1186/s12874-016-0237-9 (PMC5062833; doi:10.1186/s12874-016-0237-9)
Supplement: Additional file 4: — Boxplot of the incidence of sepsis stratified by protocol used to identify cases and on data source. (PDF 6 kb) [file 12874_2016_237_MOESM4_ESM.pdf]

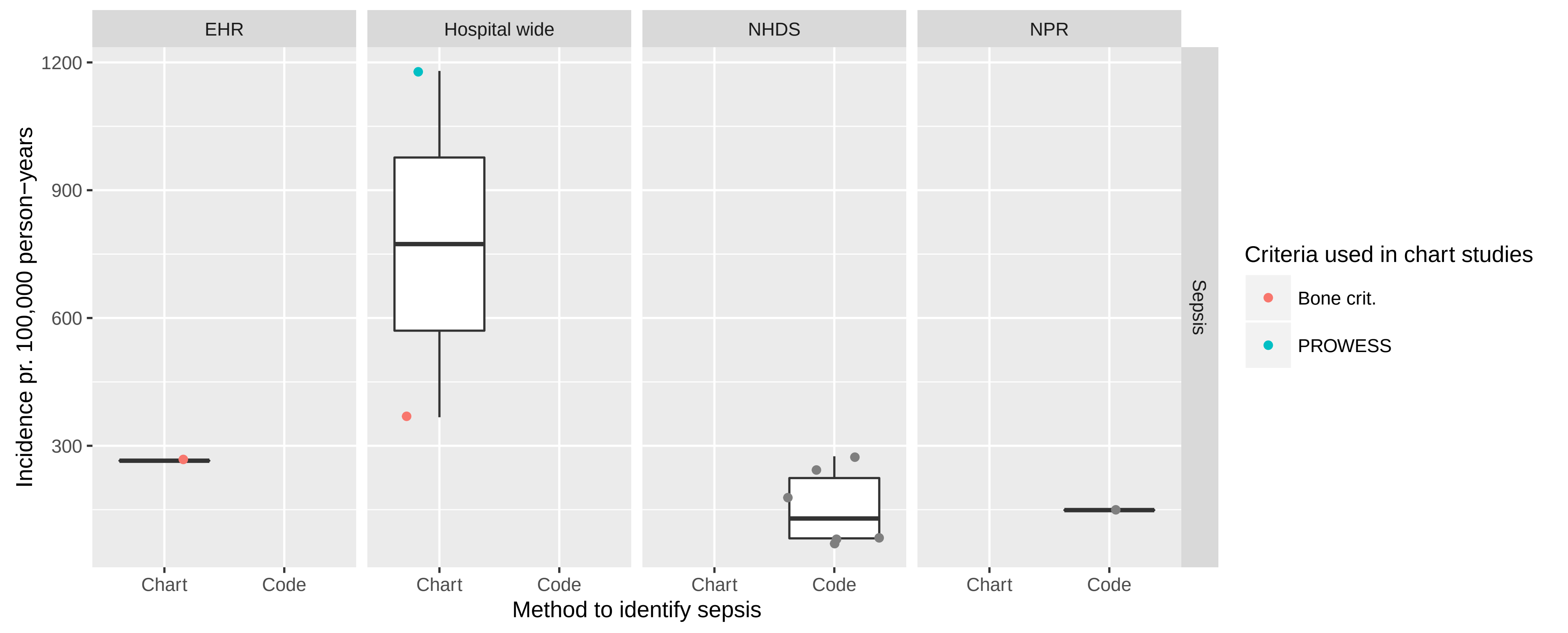
EHR
Hospital wide
NHDS
NPR
1200
900
600
300
Incidence pr. 100,000 person−years
Chart
Code
Chart
Code
Chart
Code
Chart
Code
Method to identify sepsis
Sepsis
Criteria used in chart studies
Bone crit.
PROWESS
