# Supplementary figures and images for: Differences in reported sepsis incidence according to study design: a literature review
Source: BMC Med Res Methodol. 2016 Oct 12;16:137. doi: 10.1186/s12874-016-0237-9 (PMC5062833; doi:10.1186/s12874-016-0237-9)

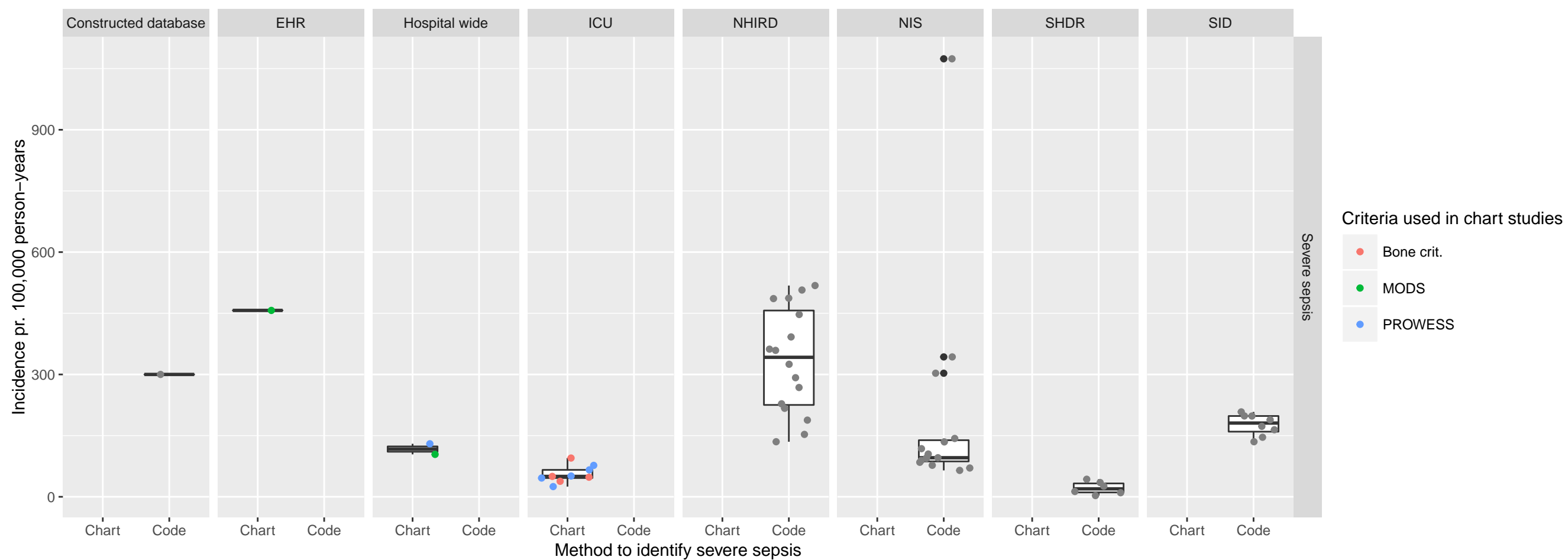

Supplement: Additional file 5: — Boxplot of the incidence of severe sepsis stratified by protocol used to identify cases and on data source. (PDF 10 kb) [file 12874_2016_237_MOESM5_ESM.pdf]
